# A 90-Day Oral Toxicity of Hydroethanolic Root Extract of *Carissa spinarum* in Wistar Rats

**DOI:** 10.1155/2021/5570206

**Published:** 2021-04-20

**Authors:** Komlan M. Dossou-Yovo, Aboudoulatif Diallo, Povi Lawson-Evi, Yendubé T. Kantati, Tchin Darré, Batomayena Bakoma, Kwashie Eklu-Gadégbéku

**Affiliations:** ^1^Laboratory of Physiology and Pharmacology, Faculty of Sciences, University of Lome, Lome, Togo; ^2^Laboratory of Toxicology, Faculty of Health Sciences, University of Lome, Lome, Togo; ^3^Laboratory of Pathological Anatomy and Cytology, Faculty of Health Sciences, University of Lome, Lome, Togo

## Abstract

**Background:**

Herbal medication is a worldwide and ancient practice, mostly in developing countries, where a large part of the population is involved in this practice. Hence, studies must be conducted to evaluate their safety and efficiency to avoid or prevent toxicological risks due to their usage. In Togo, *Carissa spinarum* is a medicinal plant belonging to Apocynaceae family, used as an aphrodisiac or to heal some ailments including malaria, sickle cell anemia, hypertension, pain, and asthma. Notwithstanding its several ethnomedicinal benefits, just a few toxicological data associated with its chronic use are available.

**Objective:**

Therefore, this study aims to assess the toxicity of an ethanolic root extract of *Carissa spinarum* in Wistar rats.

**Methods:**

The 90-day oral toxicity process following OECD TG 408 guidelines is used. Male Wistar rats received *Carissa spinarum* root hydroethanolic extract at 500 and 1000 mg/kg for 90 days by oral gavage. Body weight changes, hematological and blood biochemical parameters, organ weight changes, malondialdehyde as a lipoperoxidation marker expressed according to tissue proteins, and histopathology of vital organs were assessed.

**Results:**

No signs of toxicity or mortality were observed during the 90 days experiment. Hematological parameters have not shown any treatment-related abnormalities. According to biochemical parameters, an increase in the chloride ion level was observed at 1000 mg/kg (*p* < 0.01). There was no significant difference between the treated groups and the control group concerning the malondialdehyde concentration, body weight, and organ relative weight. No changes in necropsy and histopathology of vital organs associated with extract treatment were observed.

**Conclusion:**

The results indicated that an ethanolic root extract of *Carissa spinarum* does not cause adverse effects, which can lead to Wistar rats' death after 90-day oral administration at 500 and 1000 mg.

## 1. Introduction

*Carissa spinarum* is a spiny plant of Apocynaceae family traditionally used to heal many ailments such as sickle cell anemia, malaria, hypertension, headache, pain, asthma, and sexual weakness [1–3], particularly in Togo and in the West African region in general. Herbal medicinal healing is a worldwide and ancient practice, mostly in developing countries, where a large part of the population is involved in this practice [4]. In some south Saharan countries, this practice is maintained [5], whereas in developed countries, we observe an increase in the use of medicinal plants [6–8]. This interest for medicinal plants may be explained by many reasons such as the fact that plants are the main source of new bioactive molecules for pharmaceutical industries [9–11]. Other reasons are that plants are considered inoffensive to people [12, 13] or are simply the only or the main health care product accessible for some people [14]. This practice can be due also to financial constraints [13, 15]. Despite these numerous reasons that lead to herbal medications, caution must be taken in plant usage due to side effects such as liver damage or respiratory failure associated with continuous use of some plants [16]. Previous studies reported that *Carissa spinarum* has some pharmacological properties such as anticonvulsant effect [17], diuretic effect [1], and analgesic effect [2]. It is reported to contain some chemical groups such as tannins, saponins, flavonoids, and cardenolides [18]. Plant cardiac glycosides can be involved in case of poisoning by its action on Na^+^/K^+^ pump [19]. This can lead to severe side effects such as arrhythmias, bradycardia, and hypotension [13], which sometimes can be fatal [20, 21]. Some toxicological data available on the root hydroethanolic extract reported that its lethal dose 50 (LD50) is above 5 mg/kg and it may be relatively safe for 28-day oral repeated-dose administration in Wistar rats [18]. Nevertheless, there are no toxicological data on the chronic use of *Carissa spinarum* roots. This information may be important mainly in the use of *Carissa spinarum* in chronic diseases, healing, or life-treating ailments such as erectile dysfunction, diabetes, and hypertension. This study aims, hence, to evaluate the long-term toxicity of hydroethanolic root extract of *Carissa spinarum* by 90-day oral administration in Wistar rats to have additional toxicological data on *Carissa spinarum* root use.

## 2. Materials and Methods

### 2.1. Collection and Extraction of Plant Materials

*Carissa spinarum* roots were collected at Noépé (6°17′38″N and 1°1′19″E, Togo) and were identified at the Botany and Ecology Department of the University of Lomé. The specimen was kept at the herbarium of University of Lomé under NTOGO15579. The roots were cleaned and dried under climatization (20°C) in the Animal Physiology Laboratory of University of Lomé. Then, the dried and pulverized roots were soaked in mixed ethanol-water (50-50) for 72 h under automatic agitation on an orbital shaker (IKA KS 501Orbital Digital Platform Shaker). After double filtration with cotton and Whatman paper, the filtrate was evaporated at 45°C using a rotating evaporator (IKA RV 10 Digital).

### 2.2. Animals

Male Wistar rats weighting 161.05 g were used for the experimentations. They were provided by the Animal Physiology Department and were acclimated at least one week prior the debut of the manipulations. The animals were fed with rodent standard diets and water *ad libitum*. Animals before and during the study were kept at 22°C ± 2°C with a relative humidity level of 40% and the photoperiod was 12-h light and 12-h dark. Animal care and handling were conformed to accepted guidelines [22, 23]. Ethical approval was obtained from the institutional Ethical Committee for Teaching and Research under the number (ref no. CNCB-CEER 2801/2010).

### 2.3. Experimental Design

Repeated-dose oral toxicity of 90 days was performed according to OECD guidelines 408 [24]. Doses were assigned based on previous toxicological studies [19], and as not limited by physical-chemical nature of the extract, the highest dose was chosen with the aim to induce toxicity, not death or severe toxicity as recommended by OECD guidelines (TG 408). Animals were divided into three groups of 8 each. The first group (group 1) received distilled water and served as the control group. The second (group 2) and the third (group 3) groups received, respectively, 500 mg/kg and 1000 mg/kg body weight of *Carissa spinarum* root hydroalcoholic extract. The extract was administered daily for 90 days at the same time, and the animals were observed at least twice daily for morbidity and mortality. The body weights were recorded every day.

### 2.4. Hematological and Biochemical Parameters

On the 91st day after 12 h of fasting, the rats were anesthetized by ether. Blood samples were collected from retro-orbital sinus in dry tubes for biochemical analyses and in EDTA tubes for hematological analyses. Biochemical parameters such as alanine aminotransferase (ALAT), aspartate aminotransferase (ASAT), alkaline phosphatase, creatinine and glucose, triglyceride, and phosphocreatine kinase (CPK) were assessed. Dosage of some ion levels in serum like sodium, chloride, potassium, and calcium was also assessed. Hematological parameters achieved were white blood cell count (WBC), red blood cell count (RBC), hematocrit (HCT), hemoglobin (HB), mean corpuscular hemoglobin concentration (MCHC), mean corpuscular hemoglobin (MCH), platelet count (PLT), and mean corpuscular volume (MCV).

### 2.5. Gross Observation and Organ Weights

After exsanguination, each rat was euthanized by ether and then dissected for macroscopic examination. The liver, kidney, testicles, heart, and spleen were isolated and weighted. Relative organ weight was calculated based on the following equation: relative weight  = (weight of the organ/body weight of the animal on sacrifice day)  × 100. Samples were thereafter fixed in 10% formalin solution to achieve histopathological sections.

### 2.6. Lipid Peroxidation Test and Total Protein Assay

Lipid peroxidation test and protein assay were carried out on heart, liver, and kidney according, respectively, to Gérard-Monnier et al. [25, 26] using the 1-methyl-2-phenylindole and Bradford method [27, 28]. Malondialdehyde (MDA) concentration was expressed in nM/mg of protein.

#### 2.6.1. Lipid Peroxidation Test

The removed organs were rinsed in 9 g/L NaCl, crushed in a glass pot in the presence of Tris-HCl buffer (10 mM; pH 7.5). The reaction solution contained 650 *μ*L of 1-methyl-2-phenylindole activated by acetonitrile, 250 *μ*L of MDA or organ homogenate, 150 *μ*L of the HCl solution with the concentration of 12 N, and 10 *μ*L of BHT (butylated hydroxy toluene) solution at a concentration of 0.1 M. The tubes were incubated at 45°C for 1 hour. The tubes were then centrifuged at 1881.59 g per minute for 10 minutes. The absorbance was read at the wavelength of 586 nm. For quantitation, a calibration curve of an accurately prepared standard MDA solution was done.

#### 2.6.2. Protein Assay

The Bradford assay is based on the use of Coomassie blue (G-250). The cationic form (under acid conditions) of Coomassie blue is red, whereas the anionic form is blue. The Bradford reagent is primarily protonated and red as it is an acidified solution. Upon to protein binding, the Coomassie blue dye becomes unprotonated and blue. The change in the color is measured spectroscopically at 595 nm.

The supernatant obtained after tissue extraction was diluted at 1/20 in phosphate buffer and homogenized. Five microliters of the test sample or blank were collected in triplicate in tubes, and 250 *μ*L of Bradford reagent were added in each tube. After 30 min of incubation at room temperature, the samples were read at 595 nm.

### 2.7. Statistical Analysis

Results were expressed in mean ± standard error of the mean (SEM). Statistical analysis was performed by analysis of variance (ANOVA) with Tukey's test to evaluate the difference between two groups. Values of *p* < 0.05 were considered significant. The InStat statistical package (GraphPad Prism 6.02) was used to carry out all statistical analyses.

## 3. Results

### 3.1. Clinical Observations

The 90-day oral administration of the extract did not provoke behavioral changes in the treated groups. No death or adverse effects were observed during the experiment.

### 3.2. Body Weight and Organ Ratio Weight

Our results have shown that the extract did not induce any modification of the mean weight of rats after 90 days of administration (Table 1). Rats' organ ratio weight after 90 days of experiment did not show significant differences between groups (Table 2).

### 3.3. Hematological Parameters

No abnormality was observed on hematological parameters after 90 days of administration of *C. spinarum* (Table 3).

### 3.4. Biochemical Parameters

For biochemical parameters, no significant changes were observed. However, the electrolytes have presented some variations, principally a significant increase of chloride ions at 1000 mg/kg (Table 4).

### 3.5. Gross Observation

No abnormalities were detected after gross examination of organs between the treated and control groups. No inflammation, internal bleeding, lesions, or deformity was observed in the liver, kidney, testis, spleen, and heart in treated rats compared with the control group following the administration of *Carissa spinarum* root extract at different doses for 90 days of treatment.

The histological sections of heart, liver, kidney, testis, and spleen have not shown abnormalities in the architectural structure of organs in any group (data were not shown).

### 3.6. Lipid Peroxidation and Protein Assay

The lipid peroxidation test in the heart, liver, and kidney has not shown significant differences of malondialdehyde (MDA) concentration between control and treated groups (Table 5).

## 4. Discussion

This study was conducted to evaluate the 90-day oral toxicity of *C. spinarum*, a plant used for many purposes in Togo and the West African region mainly as an aphrodisiac [29]. Based on previous toxicological results [18], a limit test of 90-day oral toxicity test was performed at 500 and 1000 mg/kg according to OECD 408 guidelines. The *C. spinarum* hydroalcoholic root extract at 500 and 1000 mg/kg did not provoke toxic effects capable to lead treated rats to death or affect their behavior as no death or clinical abnormalities were observed.

Body weight reduction can be a sensitive and simple index of toxicity, and a 5% reduction in body weight is considered an empirical predictor of pathological findings [30]. The body weight change of the treated group rats compared with the control group had not shown significant difference after 90 days of experiment. The organ relative weight is an indicator of hypertrophy or atrophy of organs [31]. It is a well-accepted indictor of chemical effects on organs [32]. The relative weight of organs such as heart, spleen, testis, liver, and kidney has not shown significant difference between the treated groups and the control. Additionally, histological data have not shown any structural abnormalities as all architectures of the studied organs were conserved over all groups. The extract at 500 and 1000 mg/kg did not induce adverse effects on the studied organs.

Another good toxicity predictor is blood cells, which are the first cells exposed to a toxic when it reaches the systemic circulation. Hence, red blood cells give a good prediction on the toxic potential of a molecule [18, 33]. In our study, the hematological parameters of the treated groups have not shown significant difference when compared to the control group. The hydroalcoholic root extract of *C. spinarum* at 500 and 1000 mg/kg did not reveal toxic effects on blood cells [34]. The liver and kidney are key organs ensuring important role for the organism. The liver is the main organ involved in substance metabolism, and due to this, it is exposed to toxic effects. The kidney by its functional unit nephrons is involved in the excretion of waste substances and toxins in the body. Hence, a damage due to plant-related products or toxic substance ingestion by mechanisms such as tubular toxicity, crystal nephrotoxicity, or glomerular hemodynamic alterations can lead to loss of the kidney or death if appropriate medicinal therapy is not given to the patient [13]. Their exploration is therefore important in toxicity studies and can be based on some biomarker level dosage.

Located in hepatocytes, cytoplasm, and mitochondria (for ALAT), transaminases (ASAT, ALAT) associated to ALP and glucose which are found for the first in the biliary canaliculi and epithelial cells comprising the bile ducts are parameters to explore the liver toxicity [35, 36]. ASAT and ALAT are considered leakage enzymes; thus, their augmentation in blood serum may be the result of hepatocellular or mitochondrial membrane alterations following liver injury. ALP on its own is considered a cholestatic induction enzyme and is ‘‘induced” for their elevation following obstruction to bile flow [37]. Our study did not show significant differences of these biomarkers when treated groups are compared to the control group. These results are close to histological data, which did not reveal any architectural or structural abnormalities of the concerned organs.

Renal functional exploration can be based on urea, creatinine, and electrolyte dosage. They are good markers of renal function. In our study, changes in urea and creatinine were not significant. It suggests that the extract did not cause renal dysfunction. However, a significant increase in chloride ion level at 1000 mg/kg suggested a misbalance in the serum chloride level, which can be a simple result of the diuretic activity of *C. spinarum* [1]. In that case, the loss of an important volume of diluted urine may explain this hyperchloremia [3, 38, 39].

Malondialdehyde is an oxidative stress marker that results in the cell membrane disruption provoked by reactive oxidative stress attack on polyunsaturated fatty acids. Then, MDA is a product of lipid peroxidation. This lipoperoxidation derives aldehydes, which can damage the protein by forming covalent adducts. This process can lead to tissue destruction [40]. Oxidative stress is a process due to an imbalance between reactive oxygen species (ROS) and antioxidants. This process causes structural and functional damage in organs and then, it can lead to toxicity [41]. In our study, the MDA level was assessed in the heart, liver, and kidney. We did not observe significant differences according to MDA concentration when comparing control with treated groups. This result suggests that *C. spinarum* did not favor the oxidative process in the studied organs during the 90-day oral administration.

## 5. Conclusion

In this study, we have evaluated the 90-day oral toxicity of the hydroalcoholic extract of *C. spinarum* root. The results showed that at 500 and 1000 mg/kg, *C. spinarum* hydroalcoholic root extract is relatively safe for oral administration to Wistar rats at 500 and 1000 mg/kg. Nevertheless, more toxicological studies must be performed on key organs *ex vivo* or *in vitro* to confirm its real safety for chronic use, mainly on the cardiovascular system, liver, and kidney, which are important organs of our body.

## Figures and Tables

**Table 1:** Effect of the hydroethanolic extract of *Carissa spinarum* root on rats' body weight.

Weeks	Hydroethanolic root extract of *C. spinarum*	
	Control	500 mg/kg	1000 mg/kg
0	159.58 ± 8.51	163.16 ± 7.48	160.41 ± 7.73
1	160.33 ± 7.49	158.75 ± 7.65	162.91 ± 5.14
2	160.16 ± 8.41	164.50 ± 7.30	164.58 ± 5.37
3	163.00 ± 8.36	165.91 ± 8.16	165.00 ± 5.73
4	170.91 ± 8.82	171.33 ± 8.46	170.00 ± 6.25
5	172.83 ± 8.35	175.09 ± 7.78	171.63 ± 6.07
6	178.83 ± 7.87	180.54 ± 8.90	176.60 ± 7.48
7	175.58 ± 7.69	183.00 ± 9.18	176.30 ± 6.89
8	175.41 ± 7.67	186.00 ± 9.10	179.40 ± 6.54
9	175.50 ± 7.72	178.88 ± 7.84	178.70 ± 6.50
10	175.45 ± 8.55	179.62 ± 7.99	179.00 ± 6.20
11	186.50 ± 9.97	179.87 ± 9.17	177.50 ± 5.19
12	184.20 ± 10.93	189.66 ± 8.44	169.50 ± 7.65
13	188.77 ± 12.77	194.00 ± 11.26	177.75 ± 7.39

Each value is a mean ± SEM, *n* = 8.

**Table 2:** Effect of the hydroethanolic extract of *Carissa spinarum* root on organs relative weight.

Organs	Hydroethanolic root extract of *C. spinarum*	
	Control	500 mg/kg	1000 mg/kg
Liver	3.043 ± 0.147	3.075 ± 0.225	2.892 ± 0.197
Heart	0.462 ± 0.032	0.442 ± 0.037	0.418 ± 0.017
Kidney	0.626 ± 0.021	0.665 ± 0.027	0.630 ± 0.028
Spleen	0.293 ± 0.020	0.266 ± 0.036	0.284 ± 0.028
Testis	1.078 ± 0.051	1.198 ± 0.201	1.014 ± 0.102

Each value is a mean ± SEM, *n* = 8.

**Table 3:** Effect of the hydroethanolic extract of *Carissa spinarum* root on hematological parameters.

Parameters	Hydroethanolic root extract of *C. spinarum*	
	Control	500 mg/kg	1000 mg/kg
WBC, ×10^3^/*μ*L	2.18 ± 0.32	3.94 ± 0.68	3.31 ± 0.54
RBC, ×10^6^/*μ*L	5.90 ± 0.14	5.75 ± 0.29	5.93 ± 0.14
HB, g/dL	12.17 ± 0.33	11.88 ± 0.72	12.13 ± 0.32
HCT, %	31.53 ± 1.39	31.28 ± 1.23	30.53 ± 0.81
VGM, fL	51.76 ± 1.05	52.85 ± 1.07	51.42 ± 1.05
MCH, pg	20.58 ± 0.15	20.51 ± 0.21	20.45 ± 0.23
MCHC, g/dL	39.86 ± 0.52	40.87 ± 0.68	39.80 ± 0.36
PLT, × 10^5^/*μ*L	8.49 ± 0.54	8.20 ± 0.43	7.18 ± 0.63

Each value is a mean ± SEM, *n* = 8.

**Table 4:** Effect of the hydroethanolic extract of *Carissa spinarum* root on biochemical parameters.

Parameters		Hydroethanolic root extract of *C. spinarum*	
	Control	500 mg/kg	1000 mg/kg
Glucose, mmol/L	5.13 ± 0.36	5.10 ± 0.34	4.38 ± 0.32
ALAT, UI/L	40.91 ± 5.72	38.62 ± 4.29	38.95 ± 5.47
ASAT, UI/L	160.33 ± 6.20	174.35 ± 9.04	160.78 ± 6.68
ALP, UI/L	316.88 ± 31.83	352.67 ± 35.70	235.19 ± 33.75
Urea, g/L	0.49 ± 0.03	0.48 ± 0.05	0.41 ± 0.02
Creatinine, g/dL	4.29 ± 0.33	3.20 ± 025	3.50 ± 0.21
CPK	338.29 ± 31.83	339.39 ± 46.82	261.06 ± 28.35
Triglycerides, g/L	1.59 ± 0.11	1.75 ± 0.16	1.64 ± 0.09
Sodium, mmol/L	192.56 ± 2.85	173.93 ± 11.86	189.66 ± 9.81
Potassium, mmol/L	2.08 ± 0.14	1.66 ± 0.19	1.84 ± 0.18
Chloride, mmol/L	79.20 ± 3.83	87.23 ± 2.48	108.42 ± 8.30^*∗∗*^
Calcium, mg/dL	8.28 ± 0.37	7.11 ± 0.44	8.02 ± 0.38

Each value is a mean ± SEM, *n* = 8. ^*∗∗*^*p* < 0.01.

**Table 5:** Effect of the hydroethanolic extract of *Carissa spinarum* root on MDA concentration.

Organs		Hydroethanolic root extract of *C. spinarum*	
	Control	500 mg/kg	1000 mg/kg
Heart, nM MDA/mg protein	0.65 ± 0.17	0.90 ± 0.20	1.08 ± 0.20
Liver, nM MDA/mg protein	0.67 ± 0.05	0.52 ± 0.059	0.61 ± 0.04
Kidney, nM MDA/mg protein	1.19 ± 0.11	1.54 ± 0.14	1.47 ± 0.079

Each value is a mean ± SEM, *n* = 8.

## Data Availability

The data used to support the findings of this study are available from the corresponding author upon request.
